# Thermal-siphon phenomenon and thermal/electric conduction in complex networks

**DOI:** 10.1093/nsr/nwz128

**Published:** 2019-09-02

**Authors:** Kezhao Xiong, Zonghua Liu, Chunhua Zeng, Baowen Li

**Affiliations:** 1 State Key Laboratory of Precision Spectroscopy and Department of Physics, East China Normal University, Shanghai 200062, China; 2 Department of Mechanical Engineering, University of Colorado Boulder, Boulder, CO 80309, USA; 3 Institute of Physical and Engineering Sciences, Faculty of Science, Kunming University of Science and Technology, Kunming 650500, China; 4 Department of Physics, University of Colorado Boulder, Boulder, CO 80309, USA

**Keywords:** complex network, heat conduction, non-linear dynamics, thermoelectric properties

## Abstract

In past decades, a lot of studies have been carried out on complex networks and heat conduction in regular lattices. However, very little attention has been paid to the heat conduction in complex networks. In this work, we study (both thermal and electric) energy transport in physical networks rewired from 2D regular lattices. It is found that the network can be transferred from a good conductor to a poor conductor, depending on the rewired network structure and coupling scheme. Two interesting phenomena were discovered: (i) the thermal-siphon effect—namely the heat flux can go from a low-temperature node to a higher-temperature node and (ii) there exits an optimal network structure that displays small thermal conductance and large electrical conductance. These discoveries reveal that network-structured materials have great potential in applications in thermal-energy management and thermal-electric-energy conversion.

## INTRODUCTION

How to effectively navigate information, mass and energy in complex networks is of primary importance in our life, ranging from quick message transmission in communication networks and neuron networks to mass transportation in both local and global traffic networks and to energy transportation in power grids, to name just a few [1–3]. This problem has been addressed recently by Kleinberg [4] for flow in small-world networks. Optimal navigation with local knowledge and enhanced flow can be observed in such networks, for the reason that long-range interactions strongly affect the physical properties of real systems. The idea was adopted to electric flow in complex networks by Oliverira *et al.* [5], where enhanced flow properties can also be observed in small-world topologies.

In this work, we will study how heat energy will be effectively transported by long-range interaction in networks. As is well known, heat and electric energy are two fundamental energy forms used widely in our daily life. However, compared with the study of electric conduction, heat conduction is much less studied, in particular in network structures.

Indeed, the study of heat conduction in the last few decades has been mainly limited to 1D and 2D regular lattices (see reviews [6–8] and the references therein). However, realistic systems of heat conduction are generally not regular lattices, but complex networks such as the thermal devices of nanotube and nanowire networks, whose topologies are fundamentally different from the cases of 1D and 2D lattices [9–12].

**Figure 1.**
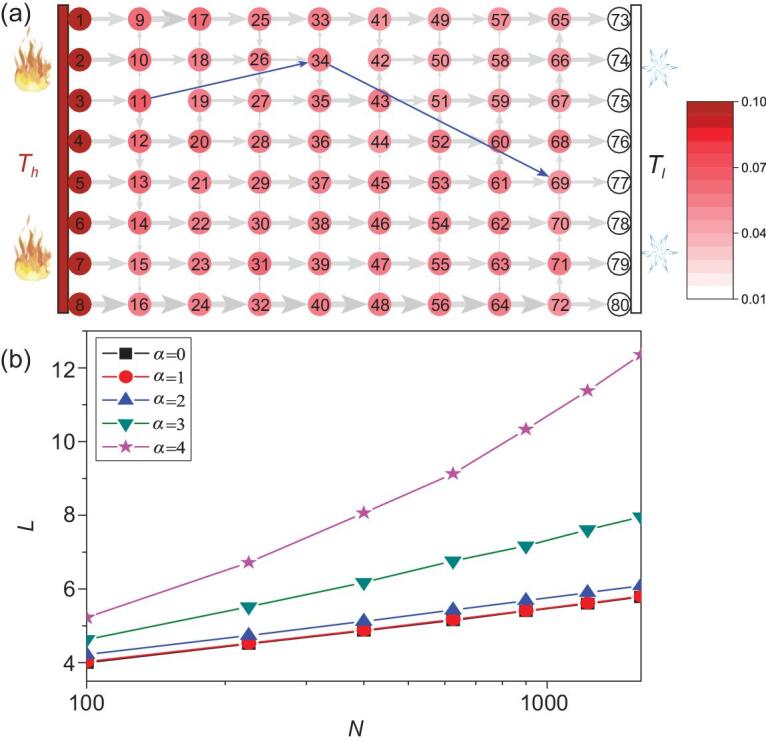
Network model of heat conduction rewired from a 2D lattice. (a) Schematic illustration of thermal transport in a rewired network from a 2D regular lattice with *m* = 8, where link11–19 and link34–42 are rewired to be link11–34 and link34–69, respectively. The *m* nodes at the most left (right) side are always contacted with the *m* source nodes in the heat bath with temperature *T_h_*(*T_l_*). Here, the solid circles denote the nodes, the colors of the nodes represent the values of the temperatures, the arrows represent the direction of the heat flows and the widths of the links denote the value of the heat flows in a stationary state. We can see clearly that the heat flow is mainly transported in the horizontal direction from left to right. However, it is surprising that the heat flow on the shortcut gets smaller than the value on the horizontal links, such as a path of heat flow 11→34→69, which is an abnormal phenomenon compared with electricity [5], meanwhile indicating that the heat conduction on the network can be seriously influenced by the network structure. (b) Influence of the parameter *α* on the average shortest distance *L* where the lines from the bottom up represent the cases of *α* = 0, 1.0, 2.0, 3.0 and 4.0, respectively.

Regarding a complex network, the regular lattices are simple and very special network structures in the sense that it has a constant degree for all nodes, whereas a complex network has distributed degrees of nodes. Moreover, there are many other different aspects between regular lattices and complex networks such as the clustering coefficient, average shortest distance and assortativity coefficient etc. [1–3], which will in turn influence the heat conduction in complex networks. In this work, we first allow the network to be embedded in a 2D physical space and then study its heat conduction by changing the network structure and coupling scheme. We find that both the network structure and coupling scheme will seriously influence the heat-conduction property; for example, they can make the network change from a heat conductor to a heat insulator. Furthermore, we reveal some specific network structures that show small thermal conductance and large electrical conductance, which provides useful information for the design of good thermoelectric materials in the sense of network structures. Specifically, we discover the phenomenon of the siphon effect in local parts of a network, where heat flux goes from a node with lower temperature to another one with higher temperature. We reveal that it is easier to observe the thermal-siphon phenomenon in a network with negative assortativity than that with positive assortativity.

## RESULTS

The physical network model of heat conduction is constructed as follows (see schematic picture in Fig. 1a). We first construct a 2D regular lattice with *N* = *m* × *m* nodes, where the degree of node*-i* is *k_i_* = 4, 3 and 2 for the inner, boundary and corner nodes, respectively. Then, for each link of the network, we randomly fix its one end and rewire the other end to a new node. Take the specific link *i*↔*j* as an example. Suppose we fix the end *i* and allow the other end *j* to be rewired to a new node-*j*′. The position of *j*′ can be chosen from other nodes except *i* by a probability ${P}_{j\hbox{'}}\sim {r}_{\mathit {jj}}^{-a}$, where ${r}_{\mathit {jj}\hbox{'}}$ is the Euclidean distance between the nodes-*j* and *j*′ [13]. Intuitively, we see that a larger *α* prefers the nodes-*j* to be chosen from the neighbors of nodes-*i* while a smaller *α* prefers the nodes-*j*′ to be chosen homogeneously from the network, which means that the length of the nanotube/nanowire between the two nodes will decrease with the increase in *α*. Therefore, the structure of the rewired network will be determined by the parameter *α*. In this way, the 2D lattice will be rewired into a complex network with different degrees at different nodes.

Figure 1b shows the dependence of the average shortest distance *L* on the network size *N* where the lines represent the cases of *α* = 0, 1.0, 2.0, 3.0 and 4.0, respectively. In fact, *L* is a key quantity to describe a complex network [1–3]. We see that *L* is proportional to log*N* for the cases of *α* < 2, indicating that it is a small-world network [1,2]. Our numerical results reveal that this property of a small world will be kept for 0 ≤ *α* ≤ 2. When *α* > 2, we have *L ∼ N^γ^*, indicating that the property of a small world is lost. Especially, it will become a random network when *α* = 0.

## Effect of rewiring

In this work, we fix *T_h_* = 0.1 and *T_l_* = 0.01. According to Eqs (8) and (9) in the Methods, we can calculate the temperatures *T_i_* for all the nodes and the heat fluxes *J_ij_* for all the links in the network. Figure 1a schematically illustrates the distribution of temperatures at nodes and heat fluxes on links in a stationary state. The solid circles denote the nodes, arrows represent the direction of heat flows, the widths of arrows denote the value of heat flows and the colors of nodes denote the value of temperatures. In Supplementary Fig. S2, we show the variety of temperature distributions of the networks with different *α*.

**Figure 2.**
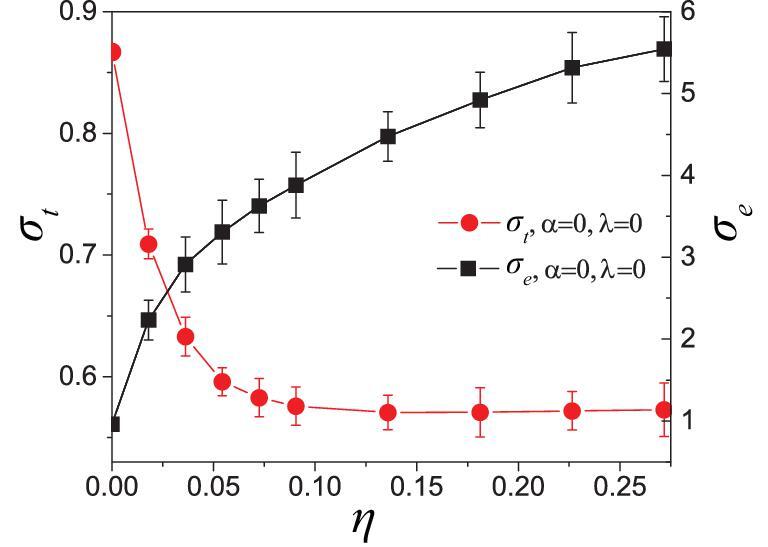
Dependence of thermal conductance *σ_t_* (left axis, red) and electrical conductance *σ_e_* (right axis, black) on rewired percentage *η*, in a randomly rewired network from a 2D regular lattice with *N* = 24 × 24.

## Thermal and electric properties of the network

The introduction of rewiring is actually a kind of set-up of long-range atomic interactions beyond the nearest neighbors. This kind of effect has been studied in regular structures. Some interesting phenomena have been found, such as that the potential extended to fourth-nearest-neighbors can be better fitted to the phonon dispersion in graphene [14], long-range interatomic forces can reduce thermal conductivity in the materials with resonant bonding [15] and diatomic lattice [16] and thermal conductivity *κ* has an interesting non-monotonic dependence with the strength of the long-range interaction in a 1D Fermi-Pasta-Ulam lattice [17]. All of these studies reveal that long-range interactions can influence the diffusion of phonons and thus can significantly affect thermal-transport properties. Here, we will investigate how the long-range interactions affect thermal transport in complex networks.

The thermal conductance of network is calculated as:

(1) \begin{equation*} {\sigma}_t={J}_t/\left({T}_h-{T}_l\right), \end{equation*}

where *J_t_* is the total heat flux of the network, i.e. the sum over all the source nodes with *T_h_* to their neighbors (see the Methods for detailed calculation). As a comparison, we also study how the rewiring links such as in Fig. 1a influence electric conduction. To this end, we let the temperatures *T_h_* and *T_l_* of the source nodes in Fig. 1a be replaced by the higher voltages *V_h_* and lower voltage *V_l_*, respectively, and let each link has a local electric conductance given by ${g}_{\mathit {ij}}={r}_{\mathit {ij}}^{-\lambda }$. Then, we can calculate the voltage *V_i_* at each node-*i* and the electric flow at each link-*l_ij_* by Kirchhoff’s law [5], and thus obtain the total electrical flow *J_e_* and electrical conductance of the network as

(2) \begin{equation*} {\sigma}_e={J}_e/\left({V}_h-{V}_l\right). \end{equation*}

To see the effect of long-range interactions, we start from a 2D regular lattice and randomly choose a certain number of links to be globally rewired. Figure 2 shows the dependence of *σ_t_* and *σ_e_* on the parameter *η*, where *η* is the percentage of the number of rewired links (to the total number of links) in a 2D regular lattice with *N* = 24 × 24. We see that, with the increase in *η*, *σ*_*t*_ decreases monotonically but *σ*_*e*_ increases monotonically, which strongly testifies that heat conduction and electric conduction are fundamentally different from each other in complex networks. Indeed, this opposite trend with an increase in *η* is because of the fact that the interfacial thermal resistance plays a more important role in reducing heat conduction, whereas, for the electric case, the influence of electric contact resistance is minimal. This result indicates that the highly rewired network might be a good candidate for thermoelectric materials that require high electric conductivity and low thermal conductivity.

Moreover, we notice clearly from Fig. 1a that, with the mainly heat transferred in the horizontal direction from left to right, it is also surprising that the heat flow on the shortcut is smaller than that on horizontal links, such as a path of heat flow 11→34→69. This is an abnormal phenomenon compared with electricity [5], indicating that the heat conduction in a network can be seriously influenced by the network structure. As is well known, the random rewiring process can create shortcuts. Watts and Strogatz have pointed out that only rewiring 10% of total links in a regular network can significantly decrease the average shortest distance of a network [13].

## Effect of the long-range coupling strength

To investigate the effect of the long-range coupling strength, we rewire all the links, namely *η* = 1, in a 2D regular network to study the effect of *α* and *λ* on heat and electric conduction.

**Figure 3.**
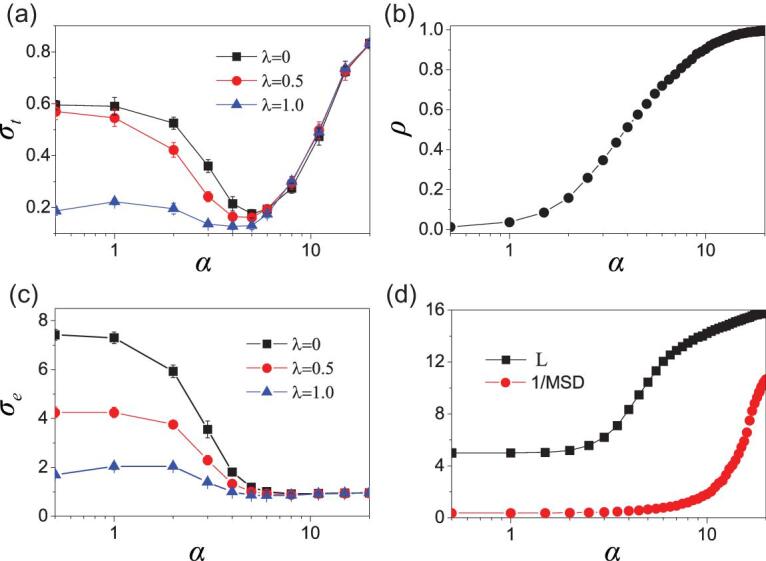
Dependence of thermal conductance and electrical conductance on the parameters *α* and *λ* for *N* = 24 × 24. (a) *σ_t_* versus *α* where the ‘squares’, ‘circles’ and ‘triangles’ represent the cases of *λ* = 0, 0.5 and 1.0, respectively. (b) The ratio of unchanged links versus *α*. (c) *σ_e_* versus *α*, where the ‘squares’, ‘circles’ and ‘triangles’ represent the cases of *λ* = 0, 0.5 and 1.0, respectively. (d) The average shortest distance and reciprocal of mean square deviation of node’s degrees versus *α*.

Figure 3a shows the dependence of *σ_t_* on the parameter *α* where the ‘squares’, ‘circles’ and ‘triangles’ represent the cases of *λ* = 0, 0.5 and 1.0, respectively. It is easy to see that, with the increase in *α*, *σ_t_* can be divided into two parts, i.e. decreasing monotonically with the increase in *α* for *α* < 5.0 but increasing monotonically with *α* for *α* > 5.0.

Notice that the rewiring process does not prohibit the original node-*j* from being chosen as the rewired node-*j*′. Thus, a larger *α* implies more chance to choose the new node-*j*′ as the original node-*j*. Let *ρ* be the probability for the original node-*j* to be chosen as the rewired node-*j*′. Figure 3b shows the dependence of *ρ* on *α*. It is interesting to see that *ρ* will be close to unity when *α* > 20, indicating that most of the rewiring processes do not happen finally.

On the other hand, we see from Fig. 3a that the three cases of *λ* = 0, 0.5 and 1.0 are quite different from each other for *α* < 5.0. For the two cases of *λ* = 0 and 0.5, *σ_t_* decreases monotonically with the increase in *α*, indicating that the network is heat conduction for smaller *α* but will become heat insulation when *α* is around 5.0. While, for the case of *λ* = 1, *σ_t_* is small for all the *α* < 5.0, implying that the case of larger *λ* is heat insulation for all the *α* < 5.0. Thus, the heat conduction can vary from heat conduction to heat insulation, depending on the network structure and coupling scheme.

To understand the bell-shaped feature of Fig. 3a, we have calculated the average shortest distance (*L*) and the mean square deviation (*MSD*) of the node’s degrees [1]. Figure 3d shows the dependence of *L* and 1/*MSD* on *α*, respectively. We see that, with the increase in *α*, both *L* and 1/*MSD* increase monotonously, while the increasing trend is different, indicating that the bell-shaped *σ_t_* in Fig. 3a comes from the competition between *L* and 1/*MSD*. When *α* < 5.0, *L* will increase rapidly but 1/*MSD* will increase relatively slowly, implying that *L* plays the leading role. The increase in *L* will enlarge the distance between high-temperature thermostats and low-temperature thermostats, and thus induce more interface resistance and reduce *σ_t_*, while, for *α* > 5.0, 1/*MSD* will increase rapidly but *L* will increase relatively slowly, implying that 1/*MSD* plays the leading role.

Later, we will show that the increase in 1/*MSD* will make the phonon pass more easily from one node to the next and thus increases *σ_t_*. It can be seen from Fig. 3a that the network is beneficial to heat conduction when *α* is 0 and 20, but insulation when *α* is around 5. These are the three most special cases. Thus, we show the dependence of thermal conductivity *κ* on size in Supplementary Fig. S1. Figure 3c shows the electrical conductance of network *σ_t_* where the ‘squares’, ‘circles’ and ‘triangles’ represent the cases of *λ* = 0, 0.5 and 1.0, respectively. Comparing Fig. 3c with Fig. 3a, we see that the former is decreased while the latter is bell-shaped, indicating that they have similar behaviors for *α* < 5.0 but fundamental differences for *α* > 5.0.

**Figure 4.**
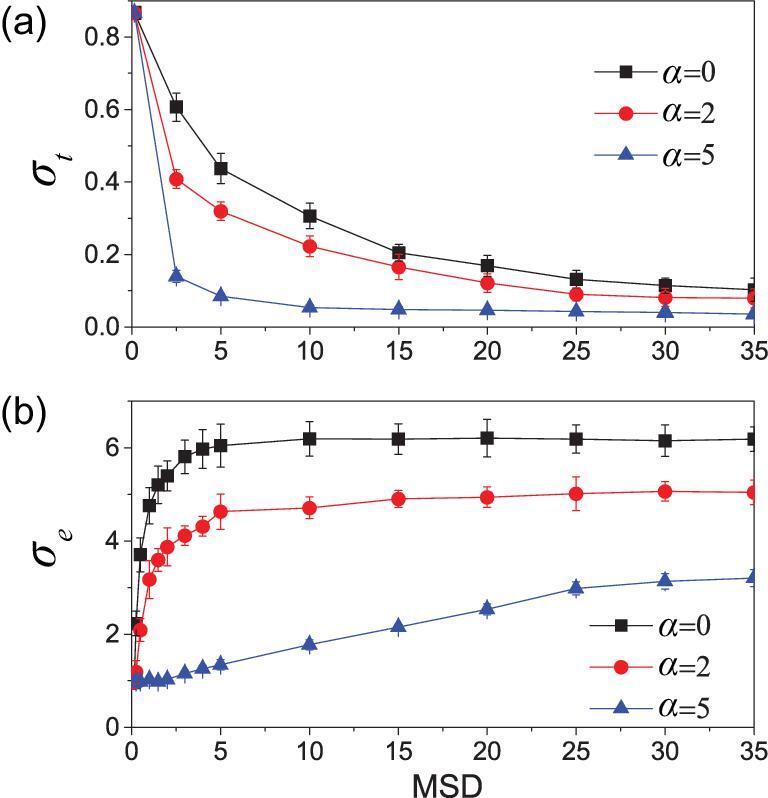
Dependence of the thermal conductance and electrical conductance on the mean square deviation (*MSD*) of the node’s degrees for *N* = 24 × 24 and *λ* = 0. (a) and (b) represent the dependence of *σ_t_* and *σ_e_* on *MSD*, respectively, where the ‘squares’, ‘circles’ and ‘triangles’ represent the cases of *α* = 0, 2 and 5, respectively.

Furthermore, we present the dependence of *σ_t_* and *σ_e_* on *MSD* in Fig. 4a and b, respectively. Here, we still adopt the method of rewiring to increase *MSD*, i.e. rewiring by the probability ${P}_{j\hbox{'}}\sim {r}_{\mathit{jj}\hbox{'}}^{-\alpha }$ as before. After each rewiring, we detect the value of *MSD*. The rewiring link will be retained if the value increases; otherwise, the rewiring link will be canceled and go back to its original site. We repeat this process until the *MSD* increases to the target value. From Fig. 4, it is easy to see that *σ_t_* monotonously decreases but *σ_e_* monotonously increases. We can also observe that *σ_t_* decreases sharply and *σ_e_* increases slowly with the increase in *α*. This finding provides good inspiration for the manufacture of thermoelectric materials from the perspective of networks.

We have so far discussed the influence of network structures on the thermal and electric properties. These results provide useful information for the design of good thermoelectric materials in the sense of network structures. In the following, we will discuss a novel heat-conduction phenomenon in complex networks: the thermal-siphon phenomenon—namely the heat ‘seems’ flow from a lower-temperature node to a higher-temperature node.

**Figure 5.**
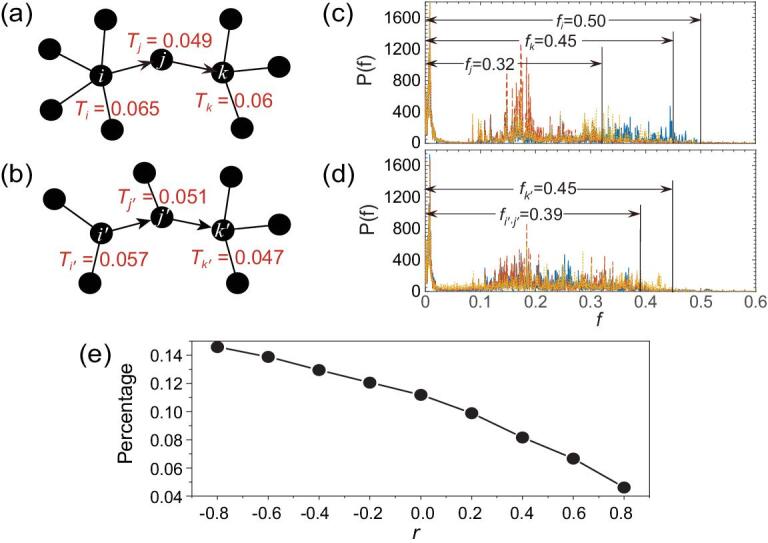
Thermal-siphon phenomenon. (a) A typical thermal-siphon phenomenon wherein heat flows seemly from a low-temperature node (*j*) to a high-temperature node (*k*). (b) A pattern of normal heat conduction. (c) and (d) represent the corresponding power spectra of the time series from the nodes-*i*, *j*, *k* and *i*′, *j*′, *k*′ in (a) and (b), respectively, where the blue solid, red dash and yellow dot lines represent the cases of the nodes*-i* and *i*′, *j* and *j*′, and *k* and *k*′, respectively. (e) Dependence of the thermal-siphon percentage on the network assortativity, which is the number of links with abnormal heat conduction divided by the total number of links in a network.

## Thermal-siphon phenomenon

We check how heat fluxes go through networks from the source nodes with *T_h_* to those with *T_l_*. For simplicity, we just chose two nodes to contact with high-temperature thermostat and low-temperature thermostat, respectively. We have checked different heat-transmission paths and found that most of the heat fluxes are transmitted from the nodes with higher temperatures to those with lower temperatures, i.e. the normal heat conduction. However, we surprisingly find that there is a small but finite probability for the heat fluxes to be transmitted from the nodes with lower temperatures to those with higher temperatures, i.e. the abnormal heat conduction that we call the thermal-siphon phenomenon.

Its typical pattern can be described by three successive nodes in a path of heat flow *i→j→k*, with temperatures *T_i_* > *T_j_* and *T_j_* < *T_k_*. Figure 5a shows such an example in the network for the case of *α* = 0 and *λ* = 0, where the temperatures for the three nodes *i→j→k* are 0.065, 0.049 and 0.060, respectively. We have confirmed this abnormal heat conduction in different network structures, indicating that it is a general phenomenon for heat conduction in complex networks. Correspondingly, we show a pattern of normal heat conduction in Fig. 5b where the temperatures for the three nodes *i′→j′→k*′ are 0.057, 0.051 and 0.047, respectively. We have confirmed that different initial temperatures do not affect the temperature of the nodes in the network at steady state. In Supplementary Fig. S3, we demonstrate that the thermal-siphon phenomenon is independent of the initial temperature of the system.

## DISCUSSION

### Effective temperatures

Does the *thermal-siphon phenomenon* really violate the second law of thermodynamics that heat always flows from a higher-temperature node to a lower-temperature node? To answer this question, we turn to the Fourier analysis of vibration at each node. In our model of heat conduction in complex network, heat is transferred in the form of vibration at a certain frequency. When heat, which consists of many phonons with different frequencies, is emitted from one node in the complex network, it will spread out in all directions and transmit to its neighbor nodes. How much heat can be transmitted through the next node depends largely on the eigen frequencies (or called spectrum) of the node. In Fig. 5c, we show the power spectra labeled as a blue solid, red dash and yellow dot lines for the nodes-*i*, *j* and *k*, respectively. We see that the widths of their spectra are different. The spectrum of the node-*j* (red) has a narrower frequency range than the spectra of the nodes-*i* (blue) and *k* (yellow). That means that, when heat flows from node-*i*, only part of it can go through *j* to *k*. Thus, only those phonons with frequencies in the spectrum of node-*j* can go through it and transmit to node-*k*.

To understand the siphon effect more quantitatively, we can introduce *effective temperatures* for the two nodes-*j* and *k* within the band of the spectrum of *T_j_*, i.e. *T′_j_* and *T′_k_* with *T*′_*j* _= *T_j_*. We notice from Fig. 5c that the amplitude of the spectrum of node-*j* (red) is larger than that of the spectrum of node-*k* (yellow) in the same frequency range, thus we have *T′_j_* > *T′_k_*. It should be noted that the effective temperature of node-*k* satisfies *T′_k_* < *T_k_* because node-*k* has more connections than node-*j* and thus has a broader spectrum.

In this sense, the energy transmission along *j→k* is still from a higher *T′_j_* to a lower *T′_k_*, implying that the second law of thermodynamics is still valid. To confirm this explanation, we show the case of normal heat conduction in Fig. 5d. We see that the spectra of the time series of *T_j_* and *T_k_* are approximately overlapped, indicating that their effective temperatures are in fact *T_j_* and *T_k_* themselves.

## Favorable network structure for the siphon phenomenon

What is the necessary condition for the siphon phenomenon? By rechecking the patterns of Fig. 5a and b, we find that the degrees for the nodes-*i*, *j* and *k* are 5, 2 and 4 in (a) and for the nodes-*i*′, *j*′ and *k*′ are 3, 3 and 4 in (b), respectively, indicating that the necessary condition is a large degree difference. To theoretically confirm it, we consider a specific case of *λ* = 0 and small *g*_4_ in Eq. (7) of the Methods. Substituting them into Eq. (7), we have:

(3) \begin{equation*} {\ddot{x}}_i=-\frac{\partial H}{\partial {x}_i}=-\frac{\partial {\sum}_i{V}_i\left({x}_i\right)}{\partial {x}_i}=\sum_{j=1}^{k_i}\left({x}_j-{x}_i\right). \end{equation*}

Equation (3) has a plane wave solution ${x}_i={Ae}^{I({q}_i{z}_i-{\omega}_i{t}_i)}.$ Substituting it back into Eq. (3), we obtain

(4) \begin{equation*} {\omega}_i=\sqrt{\sum \limits_{j=1}^{k_i}\left[1-\cos \left({q}_j{z}_j-{q}_i{z}_i+\phi \right)\right]}, \end{equation*}

where $\phi ={\omega}_i{t}_i-{\omega}_j{t}_j$ is a constant. Thus, we get the range of frequency

(5) \begin{equation*} 0<{\omega}_i<\sqrt{2{k}_i}. \end{equation*}

By *ω_i_* = 2*πf_i_*, we have $0<{f}_i<\sqrt{2{k}_i}/2\pi$. Therefore, the frequency range of node-*i* does depend on its degree *k_i_*. By Eq. (5), we have 0 < *f_i_* < 0.50, 0 < *fj* < 0.32, 0 < *f_k_* < 0.45 and $0<{f}_{i\hbox{'}}<0.39$, $0<{f}_{j\hbox{'}}<0.39$, $0<{f}_{k\hbox{'}}<0.45$ (see the arrow ranges in Fig. 5c and d, which are consistent with the spectra in Fig. 5c and d, respectively).

In a network, the degree difference of adjacent nodes is usually measured by assortativity [18].

(6) \begin{equation*} r=\frac{\left\langle {k}_i{k}_j\right\rangle -{\left\langle \left({k}_i+{k}_j\right)/2\right\rangle}^2}{\left\langle \left({k}_i^2+{k}_j^2\right)/2\right\rangle -{\left\langle \left({k}_i+{k}_j\right)/2\right\rangle}^2}, \end{equation*}

where $\langle\ \rangle$ denotes the average over all links, and *k_i_* and *k_j_* are the degrees of two connected nodes, respectively. Here, we choose an algorithm to change the assortativity [19], which has the advantage that the degree of each node will remain unchanged. The smaller the assortativity is, the greater the degree difference between adjacent nodes. We present the dependence of the thermal-siphon percentage on network assortativity *r* in Fig. 5e, which is the number of links with abnormal heat conduction divided by the total number of links in a network. The result is averaged on 20 realizations. We observe that the thermal-siphon percentage decreases with the increase in network assortativity, which is consistent with our explanation of abnormal heat conduction by Eq. (5). Moreover, we find that nodes’ temperature distribution in a network is related to the source nodes’ degree and network assortativity (see Supplementary Fig. S4).

## Further discussion of the influence of the parameter *α* on temperature distributions and thermal conductance

Equation (5) can be also used to explain the variety of temperature distributions in Fig. 1a. For the case of *α* = 0, the links will be randomly and homogeneously rewired; it is thus easy to form long-range links and this results a slight-gradient temperature distribution. With *α* increasing gradually from 0 to 5, the rewired links will prefer to connect nearby nodes, which results in the increase in *L* of a network (see black squares in Fig. 3d). Meanwhile, 1/*MSD* is small, i.e. the difference in the nodes’ degrees is relatively large (see red circles in Fig. 3d), which will lead to the non-coincidence of the phonon spectrum and thus increase the thermal resistance according to Eq. (5). Therefore, the temperature distribution gradually shows a gradient distribution and thermal conductance *σ_t_* decreases monotonically in Fig. 3a. When *α* continuously increases from 5 to 20, 1/*MSD* will gradually increase (see red circles in Fig. 3d) and thus the degree *k_i_* will gradually become the same for all the nodes. According to Eq. (5), the nodes with the same degree *k_i_* will have the same phonon spectra band, which will enable the heat flow to be transmitted more easily and thus increase the value of *σ_t_* for the part of *α* > 5.0 in Fig. 3a. Therefore, the phonon transportation becomes similar to ballistic transport, the gradient of temperature distribution gradually changes to be slight and the thermal conduction gradually increases.

## CONCLUSION

In summary, we have presented a model for heat conduction in physical networks and found that it may change from a heat conductor to a heat insulator. We reveal two phenomena. (i) An abnormal heat conduction in local parts of the network has been found, where the heat flux goes from a node with a lower temperature to another one with a higher temperature. This kind of abnormal heat conduction, which is called the ‘thermal-siphon phenomenon’, is a characteristic feature of complex networks with multiple heat paths at each node and can be understood by the concept of effective temperature. (ii) The model provides a good candidate for potential application in thermoelectric materials. The ideal thermoelectric materials are phonon glass and electric crystal, namely good electric conduction and poor thermal conduction. From the current study, we find that, for the complex network structures, as shown in Figs 2 and 4, a larger *η* and larger *MSD* are more favorable for thermoelectric application.

## METHODS

### Rewiring of a network and the FPU-*β* model of heat conduction

To study how the network structure influences heat conduction, we let each node of the complex network be an atom and the interaction between nodes can be only taken through the links. Spring-like/helical morphology is a common motif in nano-materials, which has attracted significant attention for a long time [20–24], so we let each link between two neighboring nodes be a non-linear spring to imitate the real nanotube/nanowire networks. In detail, we let the atom be the FPU-*β* model with Hamiltonian $H={\sum}_i\big[\frac{1}{2}{p}_i^2+{V}_i({x}_i)\big]$ [25,26]. The potential satisfies ${V}_i({x}_i)=\frac{1}{2}{\sum}_{j=1}^{k_i}{V}_{ij}({x}_i,{x}_j)$ with

(7) \begin{equation*} {V}_{\mathit {ij}}\left({x}_i,{x}_j\right)={c}_{\mathit {ij}}\left[\frac{1}{2}{\left({x}_j-{x}_i\right)}^2+\frac{g_4}{4}{\left({x}_j-{x}_i\right)}^4\right], \end{equation*}

where *x_i_* represents the displacement from the equilibrium position of the *i*-th atom, *c_ij_* denotes the coupling strength of link *i↔j* and the sum is for all the nearest neighbors *j* of node-*i*. In this work, we let *g*_4_ = 0.1. Further, we assume that the coupling strength decays with the increase in the distance *r_ij_* by the form ${c}_{\mathit {ij}}={r}_{\mathit {ij}}^{-\lambda }$, where the parameter *λ* represents the decaying exponent. From Eqs (7) and (9), we can obtain that the local flux *J_ij_* on link-*l_ij_* will diverge with –*λ*, i.e. local thermal conductance on link-*l_ij_* will diverge with (1 – *λ*), which is consistent with the theoretical and experimental results [27–30].

To simulate heat conduction in the network, we let the boundary nodes at the most left and right sides of the network be contacted with two heat baths of higher temperature *T_h_* and lower temperature *T_l_*, respectively. Take Fig. 1a as an example. The *m* nodes at the most left (right) side are always contacted with the *m* red (black) nodes in the bath with temperature *T_h_*(*T_l_*) and thus are considered to be the source nodes. We choose the thermal bath as the Langevin thermostat [6]. As *T_h_* > *T_l_*, there will be heat fluxes continuously from the source nodes of the most left side to the source nodes of the most right side through other nodes and links in the network. After the transient process, the network will reach a stationary state. A local temperature at each atom *i* of the network can be defined as [6–8]:

(8) \begin{equation*} {T}_i=\left\langle {p}_i^2\right\rangle, \end{equation*}

and a local flux *J_ij_* on each link-*l_ij_* can be calculated by [6,31–33]:

(9) \begin{equation*} {J}_{ij}=\left\langle {\dot{x}}_i\frac{\partial {V}_{ij}\left({x}_i,{x}_j\right)}{\partial {x}_j}\right\rangle, \end{equation*}

where $\big\langle \cdots \big\rangle$ is the time average.

## Supplementary Material

nwz128_Supplemental_File

## References

[1] Albert R , Barabasi A. Statistical mechanics of complex networks. Rev Mod Phys 2002; 74: 47–97.

[2] Boccaletti S , Latora V, Moreno Y et al. Complex networks: structure and dynamics. Phys Rep 2006; 424: 175–308.

[3] Dorogovtsev SN , Goltsev AV, Mendes JFF. Critical phenomena in complex networks. Rev Mod Phys 2008; 80: 1275–335.

[4] Kleinberg JM . Navigation in a small world. Nature 2000; 406: 845. 10972276 10.1038/35022643

[5] Oliveira CLN , Morais PA, Moreira AA et al. Enhanced flow in small-world networks. Phys Rev Lett 2014; 112: 148701. 24766030 10.1103/PhysRevLett.112.148701

[6] Lepri S , Livi R, Politi A. Thermal conduction in classical low-dimensional lattices. Phys Rep 2003; 377: 1–80.

[7] Li N , Ren J, Wang L et al. Colloquium: Phononics: manipulating heat flow with electronic analogs and beyond. Rev Mod Phys 2012; 84: 1045–66.

[8] Dhar A . Heat transport in low-dimensional systems. Adv Phys 2008; 57: 457–537.

[9] Cahill DG , Ford WK, Goodson KE et al. Nanoscale thermal transport. J App Phys 2003; 93: 793–818.

[10] Gu X-K , Wei Y-J, Yin X-B et al. Colloquium: Phononic thermal properties of two-dimensional materials. Rev Mod Phys 2018; 90: 041002.

[11] Kumar S , Murthy JY, Alam MA. Percolating conduction in finite nanotube networks. Phys Rev Lett 2005; 95: 066802. 16090972 10.1103/PhysRevLett.95.066802

[12] Pop E , Mann D, Cao J et al. Negative differential conductance and hot phonons in suspended nanotube molecular wires. Phys Rev Lett 2005; 95: 155505. 16241738 10.1103/PhysRevLett.95.155505

[13] Watts DJ , Strogatz SH. Collective dynamics of ‘small-world’ networks. Nature 1998; 393: 440–2. 9623998 10.1038/30918

[14] Tewary VK , Yang B. Parametric interatomic potential for graphene. Phys Rev B 2009; 79: 075442.

[15] Lee S , Esfarjani K, Luo T et al. Resonant bonding leads to low lattice thermal conductivity. Nat Commun 2014; 5: 3525. 24770354 10.1038/ncomms4525

[16] Han H , Feng L, Xiong S et al. Long-range interatomic forces can minimize heat transfer: from slowdown of longitudinal optical phonons to thermal conductivity minimum. Phys Rev B 2016; 94: 054306.

[17] Bagchi D . Thermal transport in the Fermi-pasta-Ulam model with long-range interactions. Phys Rev E 2017; 95: 032102. 28415308 10.1103/PhysRevE.95.032102

[18] Newman ME . Assortative mixing in networks. Phys Rev Lett 2002; 89: 208701. 12443515 10.1103/PhysRevLett.89.208701

[19] Kim BJ . Performance of networks of artificial neurons: the role of clustering. Phys Rev E 2004; 69: 045101. 10.1103/PhysRevE.69.045101 15169053

[20] Davis W , Slawson R, Rigby G. An unusual form of carbon. Nature 1953; 171: 756.

[21] Amelinckx S , Zhang X, Bernaerts D et al. A formation mechanism for catalytically grown helix-shaped graphite nanotubes. Science 1994; 265: 635–9. 17752760 10.1126/science.265.5172.635

[22] Volodin A , Buntinx D, Ahlskog M et al. Haesendonck: coiled carbon nanotubes as self-sensing mechanical resonators. Nano Lett 2004; 4: 1775–9.

[23] Shaikjee A , Coville N. The synthesis, properties and uses of carbon materials with helical morphology. J Adv Res 2012; 3: 195–223.

[24] Deng C , Pan L, Zhang D et al. A super stretchable and sensitive strain sensor based on a carbon nanocoil network fabricated by a simple peeling-off approach. Nanoscale 2017; 9: 16404–11. 29057998 10.1039/c7nr05486f

[25] Lepri S , Livi R, Politi A. Heat conduction in chains of nonlinear oscillators. Phys Rev Lett 1997; 78: 1896–9.

[26] Liu Z , Wu X, Yang H et al. Heat flux distribution and rectification of complex networks. New J Phys 2010; 12: 023016.

[27] Maruyama S . A molecular dynamics simulation of heat conduction in finite length SWNTs. Physica B 2002; 323: 193–5.

[28] Yang N , Zhang G, Li B. Violation of Fourier’s law and anomalous heat diffusion in silicon nanowires. Nano Today 2010; 5: 85–90.

[29] Chang CW , Okawa D, Garcia H et al. Breakdown of Fourier law in nanotube thermal conductors. Phys Rev Lett 2008; 101: 075903. 18764555 10.1103/PhysRevLett.101.075903

[30] Liu S , Xu X, Xie R et al. Anomalous heat conduction and anomalous diffusion in low dimensional nanoscale systems. Eur Phys J B 2012; 85: 337.

[31] Hu B , Li B, Zhao H. Heat conduction in one-dimensional chains. Phys Rev E 1998; 57: 2992–5.

[32] Hu B , Li B, Zhao H. Heat conduction in one-dimensional nonintegrable systems. Phys Rev E 2000; 61: 3828–31. 10.1103/physreve.61.3828 11088161

[33] Li B , Wang L, Casati G. Thermal diode: rectification of heat flux. Phys Rev Lett 2004; 93: 184301. 15525165 10.1103/PhysRevLett.93.184301

