# Supplementary material for: Thermal-siphon phenomenon and thermal/electric conduction in complex networks
Source: Natl Sci Rev. 2019 Sep 2;7(2):270–7. doi: 10.1093/nsr/nwz128 (PMC8288948; doi:10.1093/nsr/nwz128)
Supplement: nwz128_Supplemental_File [file nwz128_supplemental_file.docx]

**TITLE**

Thermal siphon phenomenon and thermal/electric conduction in complex networks

**AUTHORS**

Kezhao Xiong,^1,2^ Zonghua Liu,^1*^ ChunhuaZeng,^3,2^Baowen Li^2,4*^

**AFFILIATIONS**

^1^State Key Laboratory of Precision Spectroscopy and Department of Physics,

East China Normal University, Shanghai, 200062, P. R. China.

^2^Department of Mechanical Engineering, University of Colorado Boulder,

Colorado 80309 USA.

^3^Institute of Physical and Engineering Science/Faculty of Science, Kunming University of Science and Technology, Kunming 650500, P.R. China.

^4^ Department of Physics, University of Colorado Boulder, Colorado 80309 USA

*Corresponding author. Email: [zhliu@phy.ecnu.edu.cn(Z.L.);](mailto:zhliu@phy.ecnu.edu.cn(Z.L.);)

[baowen.li@colorado.edu(B.L.)](mailto:baowen.li@colorado.edu(B.L.))

**SUPPLEMENTARY MATERIALS**

**Features of the rewired network**

To check how the network structure influences heat conduction, we let each node of

complex network be an atom and the interaction between nodes can be only taken through

the links. In details, we let the atom be the FPU-$\beta$ model with Hamiltonian

[34]. The potential satisfies

$$V_{i}(x_{i})=\frac{1}{2}\sum_{j=1}^{k_{i}} c_{ij}[\frac{1}{2}(x_{j}-x_{i})^{2}+\frac{g_{4}}{4}(x_{j}-x_{i})^{4}]$$

where $x_{i}$ represents the displacement from the equilibrium position of the $i$-th atom, $c_{ij}$ denotes the coupling strength of link $i\leftrightarrow j$, and the sum is for all the nearest neighbors $j$of node-$i$. In this work, we let $g_{4}=0.1$. Further, we assume that the coupling strength decays with the increase of the distance $r_{ij}$ by the form $c_{ij}=r_{ij}^{-\lambda}$ , where the parameter $\lambda$ represents the decaying exponent.

The motion of the atoms in the rewired network satisfies the canonical equations

$\dot{x}_{i}={\partial H}/{\partial p_{i}}$;$\dot{p}_{i}=-{\partial H}/{\partial x_{i}}$. To implement the heat conduction of network, we here let

those boundary nodes at the most left and right sides of network be contacted with heat

baths with higher temperature $T_{h}$ and lower temperature $T_{l}$, respectively. We choose the

thermal bath as the Langevin thermostat [35]. The dynamical equations of the $2m$ source

nodes are $\dot{x}_{i}={\partial H}/{\partial p_{i}}$ , $\dot{p}_{i}=-{\partial H}/{\partial x_{i}}+\Gamma_{h}^{i}-\gamma p_{i}$for the left boundary nodes and

$\dot{x}_{i}={\partial H}/{\partial p_{i}}$, $\dot{p}_{i}=-{\partial H}/{\partial x_{i}}+\Gamma_{l}^{i}-\gamma p_{i}$for the right boundary nodes, where $\Gamma_{h,l}$ are the

Gaussian white noises with

$$\begin{matrix} \left\langle\Gamma_{h,l}(t)\right\rangle=0, \\ \left\langle\Gamma_{h}(t)\Gamma_{h}(0)\right\rangle=2\gamma T_{h}\delta(t), \\ \left\langle\Gamma_{l}(t)\Gamma_{l}(0)\right\rangle=2\gamma T_{l}\delta(t). \end{matrix}$$

The coupling strength is determined by the friction coefficient $\gamma$ in Langevin dynamics.

We let $\gamma=5$ in this paper. This value is within the range of $\gamma\in(1,100)$ recommended by Chen et al [36] so that a meaningful physics can be obtained. As $T_{h}>T_{l}$, there will be heat fluxes continuously from the source nodes of the most left side to the source nodes of the most right side through other nodes and links in the network. After the transient process, the network will reach a stationary state. A local temperature at each atom $i$of network can be defined as [35, 37]

$$T_{i}=\left\langle p_{i}^{2}\right\rangle$$

and a local flux $J_{ij}$ on each link-$l_{ij}$ can be calculated by [35, 38-40]

$J_{ij}=\left\langle\dot{x}_{i}\frac{\partial V}{\partial x_{j}} \right\rangle$

where $\left\langle\cdots\right\rangle$ is the time average.

Fig. S1 shows how the variation of thermal conductivity κ depends on size (20 times

average) for the case of $\lambda=0$. We can observe that the network is beneficial to heat

conduction when $\alpha$ is 0 and 20, but insulation when $\alpha$ is around 5 in Fig. 3a of main

text. Here, we present the size effect of thermal conductivity $\kappa$ for these three special

cases inFig. S1. The thermal conductivity $\kappa$ is calculated by [41]

$$\kappa=\frac{\left\langle J\right\rangle}{m\nabla T}$$

where $J$ stands for the total heat current, and the temperature gradient $\nabla T$ is along the

horizontal direction. To reduce boundary effects, $\nabla T$ is calculated by a linear fitting of

the temperature profiles in the central region (see Fig. S2), where the first and last source columns of the lattices are excluded. We can see clearly that the thermal conductivity $\kappa$satisfies a logarithmic divergent with $N$for $\alpha=20$, which is consistent with the result of reference [41].


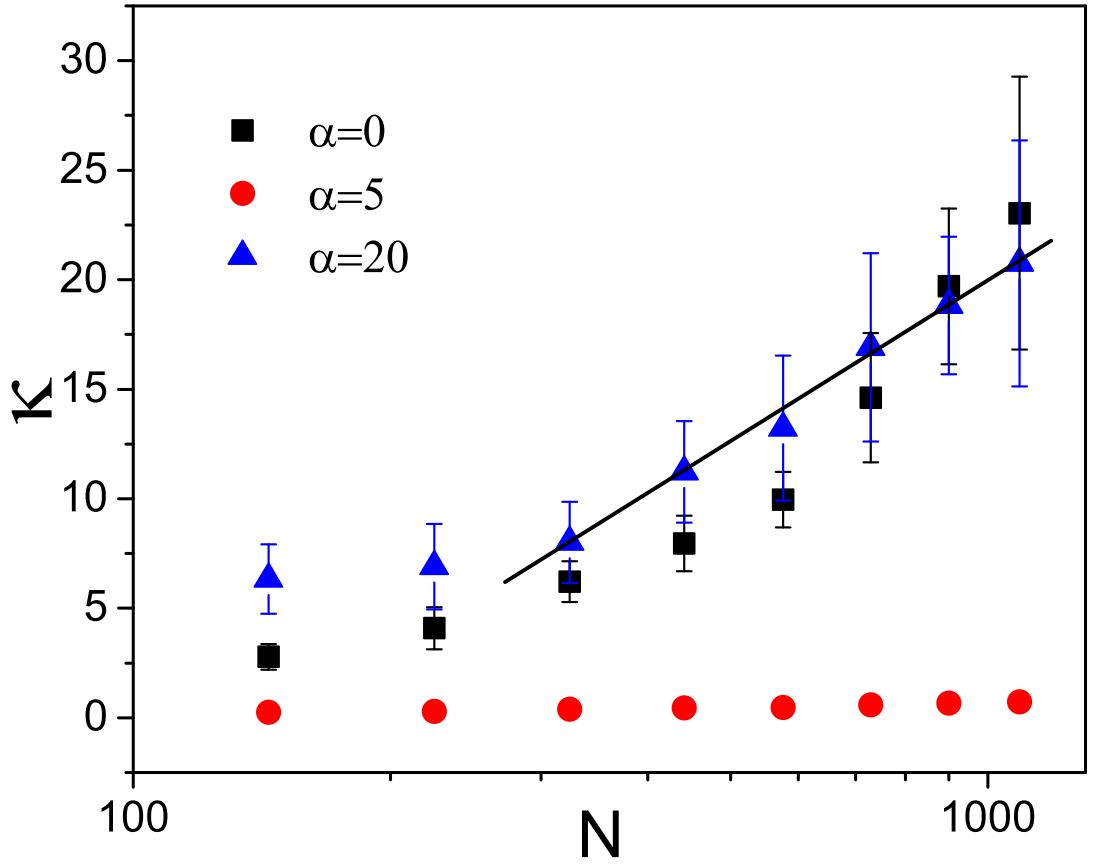


**Fig. S1.**Dependence of thermal conductivity $\kappa$ on size $N$**.**The variation of thermal conductivity $\kappa$depends on size $N$(20 times average) for the case of $\lambda=0$ where the “squares”, “circles”, and “triangles” represent the cases of $\alpha=0,5$ and 20, respectively.

In Fig. S2, we show the variety of temperature distribution of the networks with different *α*. From (*a*) to (*f*) on left column represent the cases of *α* = 0, 3, 5, 8, 11 and 20, respectively. While from (*g*) to (*l*) on right column are the averaged temperature profile corresponded to (*a*)-(*f*), respectively. *T* is the averaged value of atoms’ temperature with the same coordinate in x direction.


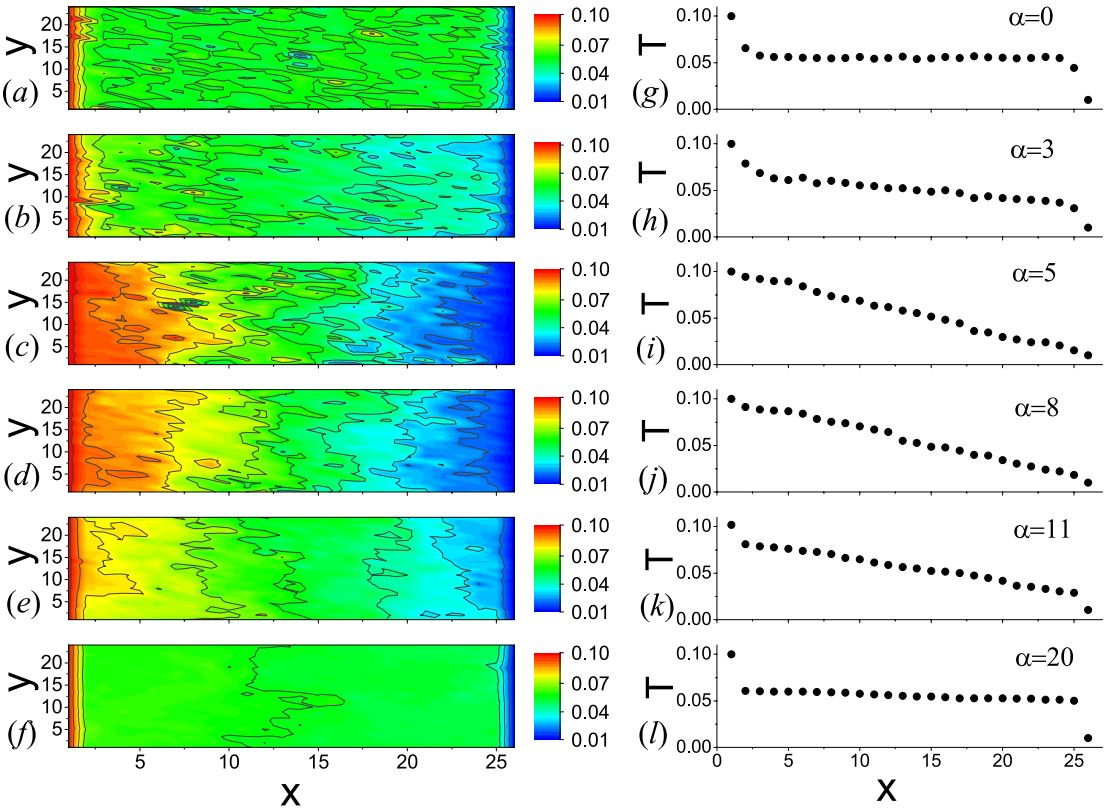


**Fig. S2.** Temperature distributions for fixed $\lambda=0$ and different $\alpha$. From (*a*) to (*f*) on left column represent the cases of $\alpha=0,3,5,8,11$ and 20, respectively. While from (*g*) to (*l*) on right column are the average temperature profile corresponded to (*a*)-(*f*), respectively. *T* is the averaged value of atoms’ temperature with the same coordinate in x direction.

In Fig. S3, we show that different initial temperatures do not affect the temperatures of the nodes in the network at steady state, which proves that the thermal siphon phenomenon is independent of the initial temperature of the system. Firstly, we set the initial temperature of the heat source same value. After the transient process, all nodes in the network reach to the initial temperature. Then, we raise the temperature of one heat source to 0.1 and lower the temperature of the other heat source to 0.01 until the system reaches the steady state. We can observe that the temperatures of nodes are almost overlapped with each other in two cases.


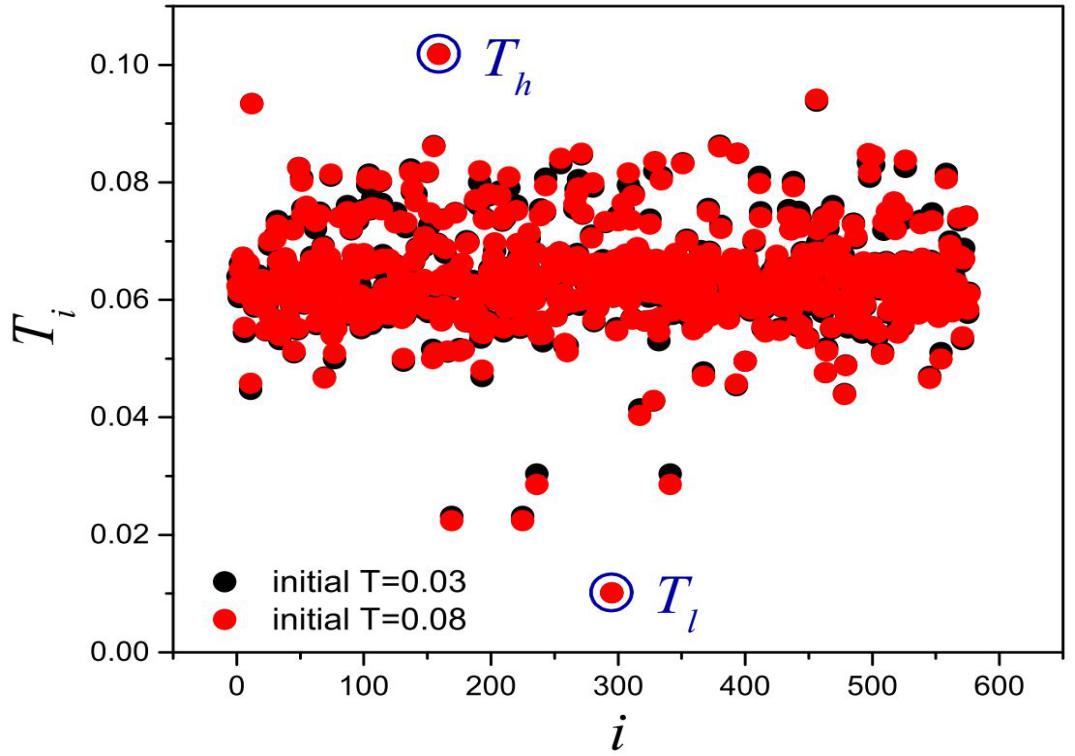


**Fig. S3.**Influence of initial temperatures of system. The temperatures of the nodes in the network at steady state from different initial temperatures of system.


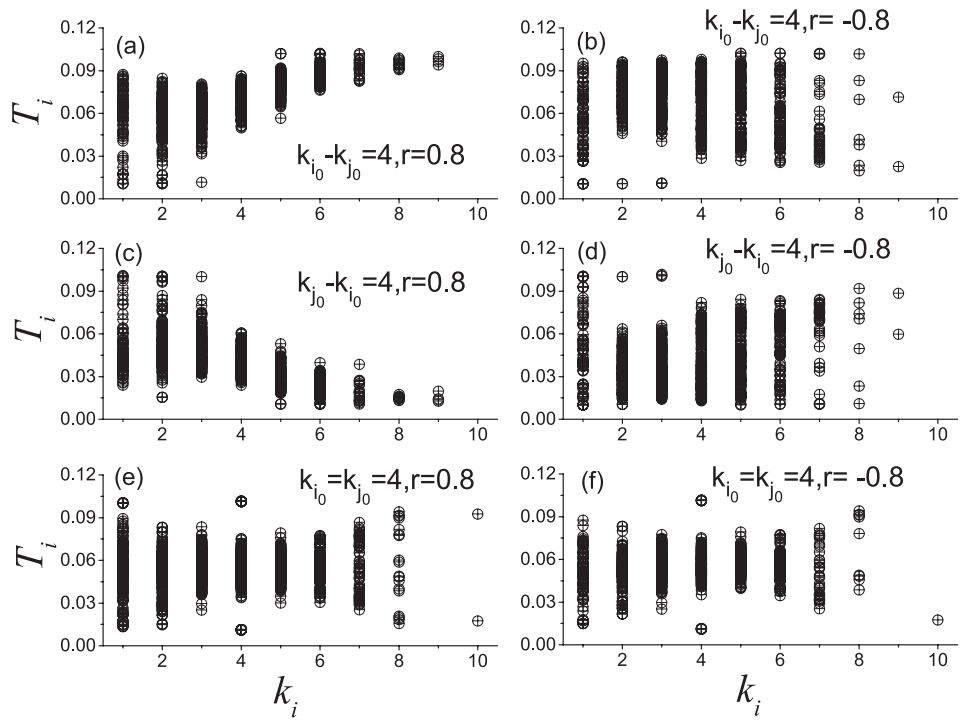


**Fig. S4.** Influence of network assortativity on the distribution of nodes temperatures. Dependence of the temperature $T_{i}$ of node $i$ on its degree $k_{i}$in the network, where $r$is network assortativity, $i_{0}$ and $j_{0}$ are high-temperature source node and low-temperature source node, respectively. The results are on 20 times statistics.

Fig. S4 shows the dependence of the temperature $T_{i}$ of node $i$ on its degree $k_{i}$ in the network, where $i_{0}$ and $j_{0}$ are the high-temperature source node and low-temperature source node, respectively. The results are on 20 times statistics. Six cases are given here. We can observe if the network has large assortativity *r* and the degree of high-temperature source node $k_{i_{0}}$ is larger than the degree of low-temperature source node $k_{j_{0}}$ such as in Fig. S4a, the temperature $T_{i}$ of node has an increasing trend with the increase of $k_{i}$ , i.e., the temperature of nodes in the network is positively correlated with the degree of nodes. On the contrary, if the network has large assortativity $r$ but the degree of high-temperature source node $k_{i_{0}}$ is smaller than the degree of low-temperature source node $k_{j_{0}}$ such as in Fig. S4c, the temperature $T_{i}$ of node has a dropping trend with the increase of $k_{i}$ , i.e., the temperature of nodes in the network is negatively correlated with the degree of nodes. In other cases, there is no obvious relationship between nodes temperature and nodes degree. Actually, both Fig. S4a and Fig. S4c are understandable. If the assortativity $r$ of the network is relatively large, the nodes with larger degree tend to be connected with each other, and the nodes with smaller degree tend to be connected with each other. According to the phonon spectrum theory in main text, phonon spectrum of the nodes with small degree difference will overlap with each other. Therefore, as a node with larger degree connects to the high-temperature source, and a node with smaller degree connects to the low-temperature source, the relationship between temperature $T_{i}$ and degree $k_{i}$ in the network will be positive. On contrary, as a node with larger degree connects to the low-temperature source, and the node with smaller degree connects to the high-temperature source, the relationship between temperature $T_{i}$ and degree $k_{i}$ in the network will be negative.

**A brief theory for electrical transition from conduction to insulation**

For the transition from electric conduction to insulation, we here give a brief theory. We

let each link of network have a local electric conductance given by $g_{ij}=r_{ij}^{-\lambda}$ , where $r_{ij}$

is the Euclidean distance between nodes $i$ and $j$, and the parameter $\lambda$ represents the

decaying exponent. The adjacency matrix of network is $G=(g_{ij})$ with $g_{ij}$ being the

electric conductance. Let $g_{ii}=0$ for avoiding self-connection. Based on the condition that

the nodes at the most left (right) side have the same voltage $V_{h}(V_{l})$, for convenience, we

let them to be combined together to form the source node $i_{0}(j_{0})$. Denote $E$as the voltage

vector of *N* components. Thus, the voltage vector $E$can be expressed as

$E={(V_{h,}V_{l},V_{3,}\cdots,V_{N})}^{T}$, and can be divided into block-1 with $E_{1}=(V_{h},V_{l})^{T}$ and block-2

with $E_{2}={(V_{3},\ldots,V_{N})}^{T}$, namely

$$E=\left(\begin{matrix} E_{1} \\ E_{2} \end{matrix}\right)$$

The discrete Laplace operator on the network is , where $k_{i}^{*}=\sum_{j=1}^{N} g_{ij}$.

Correspondingly, the matrix $L$ can be divided into four submatrices $L_{11},L_{12},L_{21}$,

and $L_{22}$. According to Ohm’s law, we have

$$LE=J$$

where $J$is the external electric flow vector. According to the Kirchhoff’s law, no net

electric flow should occur except the nodes with the higher and lower voltages, namely,

$J=0$for the nodes $i=3,\ldots,N.$ Hence we have

$$\left(\begin{matrix} L_{11} & L_{12} \\ L_{21} & L_{22} \end{matrix}\right)\left(\begin{matrix} E_{1} \\ E_{2} \end{matrix}\right)=\left(\begin{matrix} J \\ 0 \end{matrix}\right)$$

From equation (8), we can obtain

$$\begin{matrix} L_{11}E_{1}+L_{12}E_{2}=J, \\ L_{21}E_{1}+L_{22}E_{2}=0 \end{matrix}$$

In order to calculate the voltages $E_{2}$ of other nodes on the network, the key procedure is

to solve equation (9). We here use the Green’s function to solve this equation [42]. Let us

introduce a matrix $P=D^{-1}G$, where $D$ is the diagonal degree matrix. It will have an

eigenvalue one with the right eigenvector being a column of 1

$\left| u^{0} \right\rangle={(1,1,\cdots,1)}^{T}$

and with left eigenvector being

$\left\langle v^{0} \right|=(\frac{k_{1}^{*}}{k^{*}},\frac{k_{2}^{*}}{k^{*}},\cdots,\frac{k_{N}^{*}}{k^{*}},)$

where $k^{*}=\sum_{i=1}^{N} k_{i}^{*}$ . Note that with this notation, we have $\langle v^{0}|u^{0}\rangle=1$.Then, we can

decompose $P$ into

$$P=M+|u^{0}\rangle\langle v^{0}|$$

in which

$$M|u^{0}\rangle\langle v^{0}|=|u^{0}\rangle\langle v^{0}|M=0$$

Further, the spectral radius of $M$ is now guaranteed to be smaller than 1, and thus$(I-M)$

is invertible with $(I-M)^{-1}=\sum_{m=0}^{\infty} M^{m}$. We rewrite equation (8) as

$$(I-M)\left(\begin{matrix} E_{1} \\ E_{2} \end{matrix}\right)=\left(\begin{matrix} J \\ 0 \end{matrix}\right)+|u^{0}\rangle\langle v^{0}|\left(\begin{matrix} E_{1} \\ E_{2} \end{matrix}\right)=\left(\begin{matrix} J \\ 0 \end{matrix}\right)+K(E)|u^{0}\rangle$$

with

$$K(E)=\langle v_{1}^{0}|E_{1}\rangle+\langle v_{2}^{0}|E_{2}\rangle$$

where $\langle v_{1}^{0}|$ represents a row vector whose components are obtained from the row

vector $\langle v^{0}|$ with component labels corresponding to the higher voltage and lower voltage,

and $\langle v_{2}^{0}|$ represents a row vector that is the remainder of $\langle v^{0}|$ after removing the

components whose labels correspond to the higher voltage and lower voltage.

Notice $M|u^{0}\rangle=0$. Upon multiplying *Ω =* (*I -M*)^-1^ to both side of equation (14) we

have

$$\left(\begin{matrix} E_{1}-K(E)u_{1}^{0} \\ E_{2}-K(E)u_{2}^{0} \end{matrix}\right)=\left(\begin{matrix} \Omega_{11} & \Omega_{12} \\ \Omega_{21} & \Omega_{22} \end{matrix}\right)\left(\begin{matrix} J \\ 0 \end{matrix}\right)$$

Further, we have

$$\begin{matrix} E_{1}-K(E)u_{1}^{0}=\Omega_{11}J, \\ E_{2}-K(E)u_{2}^{0}=\Omega_{21}J \end{matrix}$$

Consequently, we may write *E*_2_ in the following form

$$|E_{2}\rangle=K(E)|u_{2}^{0}\rangle+\Omega_{21}\Omega_{11}^{-1}|E_{1}\rangle-K(E)\Omega_{21}\Omega_{11}^{-1}|u_{1}^{0}\rangle$$

From equation (15) and (18), we obtain

$$K(E)=\frac{\langle v_{1}^{0}|E_{1}\rangle+\langle v_{2}^{0}|\Omega_{21}\Omega_{11}^{-1}|E_{1}\rangle}{1-[\langle v_{2}^{0}|u_{2}^{0}\rangle-\langle v_{2}^{0}|\Omega_{21}\Omega_{11}^{-1}|u_{1}^{0}\rangle]}$$

Similarly, $|u_{1}^{0}\rangle$ is a column vector whose components are obtained from the column

vector $|u^{0}\rangle$ with component labels corresponding to the nodes with the higher and lower

voltages; while $|u_{2}^{0}\rangle$ represents a column vector that is the remainder of $|u^{0}\rangle$ after

removing the components whose labels correspond to the nodes with the higher and lower

voltages. From equation (19), we can obtain the voltage value $V_{i}$ of each node $i$ in the

network. Then, we can calculate the *J_e_* which is the total electric flow of network.

**REFERENCES**

34. Lepri S, Livi R and Politi A. Heat Conduction in Chains of Nonlinear Oscillators.

*Phys Rev Lett* 1997; **78**: 1896-1899.

35. Lepri S, Livi R and Politi A.Thermal conduction in classical low-dimensional

lattices. *Phys Rep* 2003; **377**: 1-80.

36. Chen J, Zhang G and Li B. Molecular Dynamics Simulations of Heat Conduction in

Nanostructures: Effect of Heat Bath. *J Phys Soc Jpn* 2010; **79**: 074604.

37. Dhar A. Heat transport in low-dimensional systems. *Adv Phys* 2008; **57**: 457-537.

38. Hu B, Li B and Zhao H. Heat conduction in one-dimensional chains. *Phys Rev E*

1998; **57**: 2992-2995.

39. Hu B, Li B and Zhao H. Heat conduction in one-dimensional nonintegrable systems.

*Phys Rev E* 2000; **61**: 3828-3831.

40. Li B, Wang L and Casati G.Thermal Diode: Rectification of Heat Flux. *Phys Rev*

*Lett* 2004; **93**: 184301.

41. Wang L, Hu B and Li B.Logarithmic divergent thermal conductivity in two-

dimensional nonlinear lattices. *Phys Rev E* 2012; **86**: 040101.

42. Zhang YC, Blattner M and Yu YK. Heat conduction process on community

networks as a recommendation model. *Phys Rev Lett* 2007; **99**: 154301.
